# Southern Surgical and Gynecological Association

**Published:** 1890-10

**Authors:** 


The Southern Surgical and Gynecological Association.—The preliminary programme of the session of this association, to be held in Atlanta, Ga., November 11, 12, and 13, prox., has been received.

The President is Dr. Geo. J. Engelmann of St. Louis; Vice-Presidents, B. E. Hadra, of Texas, and Duncan Eve, of Tennessee; Secretary, W. B. Davis, of Alabama. The programme embraces thirty papers to be read, as far as announced, some of them by very distinguished men. A cordial invitation is extended to the medical profession to attend.

Complaint is made that Texas has not her full quota of representatives in this association, and Texas surgeons are urged to attend and to contribute papers, in order that our State may keep up its reputation.

